# Groundwater Depth Affects Phosphorus But Not Carbon and Nitrogen Concentrations of a Desert Phreatophyte in Northwest China

**DOI:** 10.3389/fpls.2018.00338

**Published:** 2018-03-15

**Authors:** Bo Zhang, Xiaopeng Gao, Lei Li, Yan Lu, Muhammad Shareef, Caibian Huang, Guojun Liu, Dongwei Gui, Fanjiang Zeng

**Affiliations:** ^1^State Key Laboratory of Desert and Oasis Ecology, Xinjiang Institute of Ecology and Geography, Chinese Academy of Sciences, Urumqi, China; ^2^Cele National Station of Observation and Research for Desert-Grassland Ecosystems, Cele, China; ^3^Key Laboratory of Biogeography and Bioresource in Arid Zone, Chinese Academy of Sciences, Urumqi, China; ^4^University of Chinese Academy of Sciences, Beijing, China; ^5^Department of Soil Science, University of Manitoba, Winnipeg, MB, Canada

**Keywords:** ecological stoichiometry, desert phreatophyte, *Alhagi sparsifolia*, groundwater depth, leguminous plant, plant growth strategy

## Abstract

Ecological stoichiometry is an important aspect in the analysis of the changes in ecological system composition, structure, and function and understanding of plant adaptation in habitats. Leaf carbon (C), nitrogen (N), and phosphorus (P) concentrations in desert phreatophytes can be affected by different depths of groundwater through its effect on the adsorption and utilization of nutrient and plant biomass. We examined the biomass, soil organic C, available (mineral) N, and available P, and leaf C, N, and P concentrations of *Alhagi sparsifolia* grown at varying groundwater depths of 2.5, 4.5, and 11.0 m in 2015 and 2016 growing seasons in a desert-oasis ecotone in northwest China. The biomass of *A. sparsifolia* and the C, N, and P concentrations in soil and *A. sparsifolia* showed different responses to various groundwater depths. The leaf P concentration of *A. sparsifolia* was lower at 4.5 m than at 2.5 and 11.0 m likely because of a biomass dilution effect. By contrast, leaf C and N concentrations were generally unaffected by groundwater depth, thereby confirming that C and N accumulations in *A. sparsifolia* were predominantly determined by C fixation through the photosynthesis and biological fixation of atmospheric N_2_, respectively. Soil C, N, and P concentrations at 4.5 m were significantly lower than those at 11.0 m. Leaf P concentration was significantly and positively correlated with soil N concentration at all of the groundwater depths. The C:N and C:P mass ratios of *A. sparsifolia* at 4.5 m were higher than those at the other groundwater depths, suggesting a defensive life history strategy. Conversely, *A. sparsifolia* likely adopted a competitive strategy at 2.5 and 11.0 m as indicated by the low C:N and C:P mass ratios. To our knowledge, this study is the first to elucidate the variation in the C, N, and P stoichiometry of a desert phreatophyte at different groundwater depths in an arid ecosystem.

## Introduction

In ecological stoichiometry, relationships among multiple key elements in different ecological systems are examined (Elser et al., 2000; Cross et al., 2005; Rong et al., 2015; Zhan et al., 2017). Leaf ecological stoichiometry, especially carbon (C), nitrogen (N), and phosphorus (P) stoichiometry, is an important aspect in the analysis the changes in concerned communities and ecological system compositions, structures and functions (Han et al., 2005; Venterink and Güsewell, 2010; Gao et al., 2013; Rong et al., 2015; Li et al., 2017; Zeng et al., 2017). Biologically, C, N, and P are vital chemical elements and fundamental substances for plant growth (Sterner, 1995; Elser et al., 1996; Vrede et al., 2004; Wang et al., 2015; Butler et al., 2017). C remains relatively stable at 50% of dry mass composition and provides a structural basis for plants. N is an essential proteins element that plays an important role in enzyme activities. P participates in energy transfer in plant cells. N and P are crucial basic elements in nucleic acids (Marschner, 2012; Yan et al., 2016). C, N, and P concentrations generally change with plant growth because plant differ in photosynthetic capability, nutritional requirement, and adaptation to their environment (Elser et al., 1996; Rong et al., 2015; Wang et al., 2015; Zeng et al., 2017).

C, N, and P are coupled in terms of their biochemical functions in plants. C:N and C:P mass ratios are important indicators that represent the growth status and metabolic characteristics of plants and reflect the capacity of photosynthetic C fixation when N and P are absorbed simultaneously (Wang et al., 2015). Rong et al. (2015) indicated that C:N and C:P mass ratios can represent the balance between competitive and defensive life strategies in plants. High N and P concentrations in plants likely result in low C:N and C:P mass ratios, suggesting that plants adopt a competitive strategy with a high photosynthetic rate. On the contrary, high C:N and C:P mass ratios imply that plants adopt a strong defense life strategy with a low photosynthetic rate (Sterner and Elser, 2002; Wright et al., 2004; Poorter and Bongers, 2006; Shipley et al., 2006; Rong et al., 2015). For example, the leaf C:P mass ratio of a desert plant *T. chinensis* is positively related to soil salt concentration, suggesting that *T. chinensis* uses a defensive life history strategy under salt stress with high C:N and C:P mass ratios (Rong et al., 2015; Wang et al., 2015; Zeng et al., 2017).

N:P mass ratio represents a dynamic balance between soil nutrients and plant nutrition demands and indicates the nutrient limitation of plants at a community level (Koerselman and Meuleman, 1996; Aerts and Chapin, 1999; Güsewell, 2004; He et al., 2008; Nikolic et al., 2014; Rong et al., 2015). For example, in a habitat exposed to salt stress, the leaf N:P mass ratio of *T. chinensis* is less than 14, indicating that the growth of *T. chinensis* is limited by N at the beginning of the growing season; however, the leaf N:P mass ratio is greater than 16, suggesting that the P limitation at the middle and final growing seasons (Rong et al., 2015). Therefore, C:N, C:P, and N:P mass ratios are important indicators of the efficiencies of C fixation and nutrient utilization in plants. Thus, investigations on the variations in C, N, and P and C:N, C:P, and N:P mass ratios in plants help elucidate plant growth strategy and adaptability to changes in environments and habitats and thus provide a basis for the development of strategies and policies to facilitate ecological conservation and environmental protection (Rong et al., 2015; Wang et al., 2015; Zeng et al., 2017).

The ecological stoichiometry of a specific plant species and its adaptation to extreme environments have been widely investigated. Variations in leaf N and P concentrations at genus or species levels have also been examined (Reich and Oleksyn, 2004; Kang et al., 2011; Wu et al., 2012; Rong et al., 2015; Zeng et al., 2017). Many studies on ecological stoichiometry have been conducted in the presence of different environmental factors, such as nutrients, light, prescribed burning, and plantation age (Herbert et al., 2003; Striebel et al., 2008; Xing et al., 2013; Butler et al., 2017; Zeng et al., 2017). Li W. et al. (2013) and Li W. et al. (2015) also found that water depth gradients remarkable affected the C, N, and P concentrations and mass ratios of macrophytes in Lake Erhai.

The ecological stoichiometry of terrestrial plants, especially desert phreatophytes with deep root systems, at a reduced groundwater depth in arid regions has been rarely reported. In the arid region of southwestern Xinjiang in China, groundwater table gradually reaches deeper layers in desert-oasis transition ecotone because of intensive human intervention, indicating a shortage of water resources (Liu, 2007). The decline of groundwater depth affects plant physiological adaptations, such as biomass accumulation, photosynthesis, and water use efficiency, thereby influencing species composition and community structure in ecosystems (Nilsson et al., 1997; Rood et al., 2000; Horton et al., 2003; Imada et al., 2008; Li et al., 2010; Chen et al., 2011; Gui et al., 2013; Li C.J. et al., 2015).

As one of the dominant phreatophytes in an arid desert ecosystem, *Alhagi sparsifolia* Shap. is a spiny herb that belongs to Fabaceae family and grows in China, West Asia, America, North Africa, and Mediterranean regions (Zeng and Liu, 2012). *A. sparsifolia* is a dominant perennial species that stabilizes sand dunes, prevents land erosion in arid and semiarid regions between oasis and deserts, and supports a fragile desert ecosystem (Zeng and Liu, 2012; Li C.J. et al., 2015). *A. sparsifolia* has a high crude protein concentration and serves as a major food source for herds in an oasis foreland. Therefore, *A. sparsifolia* is a vital food of the local farmers’ large livestock; thus it has a remarkable economic value (Li H.F. et al., 2013; Zhang et al., 2018).

The deep root system of *A. sparsifolia* can reach groundwater, which is a major source of water and nutrients (Arndt et al., 2004; Zeng et al., 2006). In our previous studies, the decline in groundwater depth affects the leaf physiological parameters, root characteristics, clonal growth, and propagation traits of *A. sparsifolia* (Liu et al., 2009; Zhang et al., 2011; Gui et al., 2013; Zeng et al., 2013; Peng et al., 2014). Therefore, in the present study, we hypothesized that the depth of groundwater would have a major effect on the ecological stoichiometry of *A. sparsifolia* through its influence on the absorption and utilization of nutrients, namely, C, N, and P.

## Materials and Methods

### Study Area

The study site was located at the Cele National Station of Observation and Research for Desert and Grassland Ecosystem (Cele Station; 37°00′56.37″ N, 80°43′81″ E) at the southern rim of the Taklamakan Desert in the Xinjiang Uygur Autonomous Region of China. This station is found at the desert-oasis transition ecotone, which is characterized by a 5–10 km belt of sparse phreatophytic species, dominated by *A. sparsifolia* (Zeng et al., 2013). The groundwater depth in this area ranges from 2.5 to 15.0 m. The study site has an elevation of 1366 m (asl), a mean annual temperature of 11.9°C, a mean annual potential evaporation of 2600 mm, and a mean annual precipitation of only 35 mm (Gui et al., 2013; Liu et al., 2016). Extreme temperatures can reach -31°C in winter and 49°C in summer. Thus, the climate is arid, with hot and dry summers and cold and dry winters. Soils in this area are sandy with a bulk density of 1.35 g/cm^3^ (Liu et al., 2013, 2016).

Three sites with varying groundwater depths were selected: 2.5 (37°01′18″ N, 80°42′29″ E), 4.5 (37°00′40″ N, 80°42′13″ E), and 11.0 m (37°00′33″ N, 80°42′25″ E). Each site has a large area of approximately 2 hectares. At each sampling stage, three plots with an area of 9 m^2^ were selected for each groundwater depth. In the experimental period from June to October in 2015 and 2016, samplings were conducted monthly. Therefore, total 30 plants were sampled over time in each plot and total 45 samplings were performed in each year. In this study, a large plot area and a replicate number were enough to minimize the effect of research location difference. As such, a typical randomized complete block design was not used. This experimental design is common in similar ecological studies investigating the adaptability of plants to adverse environments (Wang et al., 2015; Zeng et al., 2017). Precipitation at the Cele Station was 34.2 mm in 2015 and 43.4 mm in 2016. The average temperatures from June to October were 21.4°C in 2015 and 21.3°C in 2016. The groundwater depths in the three sites did not significantly fluctuate between 2015 and 2016.

### Field Sampling and Chemical Analysis

At each sampling, three *A. sparsifolia* plants with similar canopy sizes, heights, and stem numbers were selected from each plot. Mature leaves with similar sizes were collected. Leaf samples from four directions and different parts were gathered though inquartation (Rong et al., 2015). The leaf samples were oven dried at 105°C for 15 min, and then at 70°C to a constant weight. All of the leaves were subsequently ground using a miller and sieved through a 1 mm mesh screen for chemical element analysis. The total organic C concentration was extracted with 0.8 mol/L K_2_C_r_2O_7_ and determined using a modified Mebius method (Nelson and Sommers, 1982). The leaf N concentration was extracted with 10 mol/L NaOH and identified using Kjeldahl acid digestion (Sparks et al., 1996). The leaf P concentration was obtained through using colorimetric analysis after the samples were digested with 0.008 L H_2_SO_4_ (Thomas et al., 1967).

In a previous study, we observed no significant difference in the soil organic C, total and available N, and P concentrations below 50 cm soil depth around *A. sparsifolia* roots at 2.5, 4.5, and 11.0 m groundwater depths at the same research sites (Liu, 2011). However, soil organic C, total and available N, and P concentrations at a soil depth above 20 cm were significantly different at various different groundwater depths. Therefore, each soil sample around the canopy of *A. sparsifolia* was collected at only 0–20 cm depth in accordance with standardized collection protocols in this research from June to October. Nine soil samples were collected at each groundwater depth in each month, air dried, ground, and passed through a 2 mm sieve. Soil organic C was determined through digestion 0.8 mol/L K_2_Cr_2_O_7_ (Nelson and Sommers, 1975). Soil available (mineral) N was determined by a continuous flow analyzer after extraction with 2 M KCl solution was conducted (Li et al., 2011). Soil available P was determined by colorimetrically after extraction with 0.5 M NaHCO_3_ solution was performed (Olsen et al., 1954).

### Data Analysis

Turkey’s test was conducted for multiple comparisons of treatment means. Pearson correlation analysis was carried out to analyze the correlations of the leaf C, N, and P stoichiometry of *A. sparsifolia* and the corresponding soil C, N, and P stoichiometry. All of the analyses were performed with SPSS 19.0.

## Results

### Biomass of *A. sparsifolia* Affected by Groundwater Depth

**FIGURE 1:**
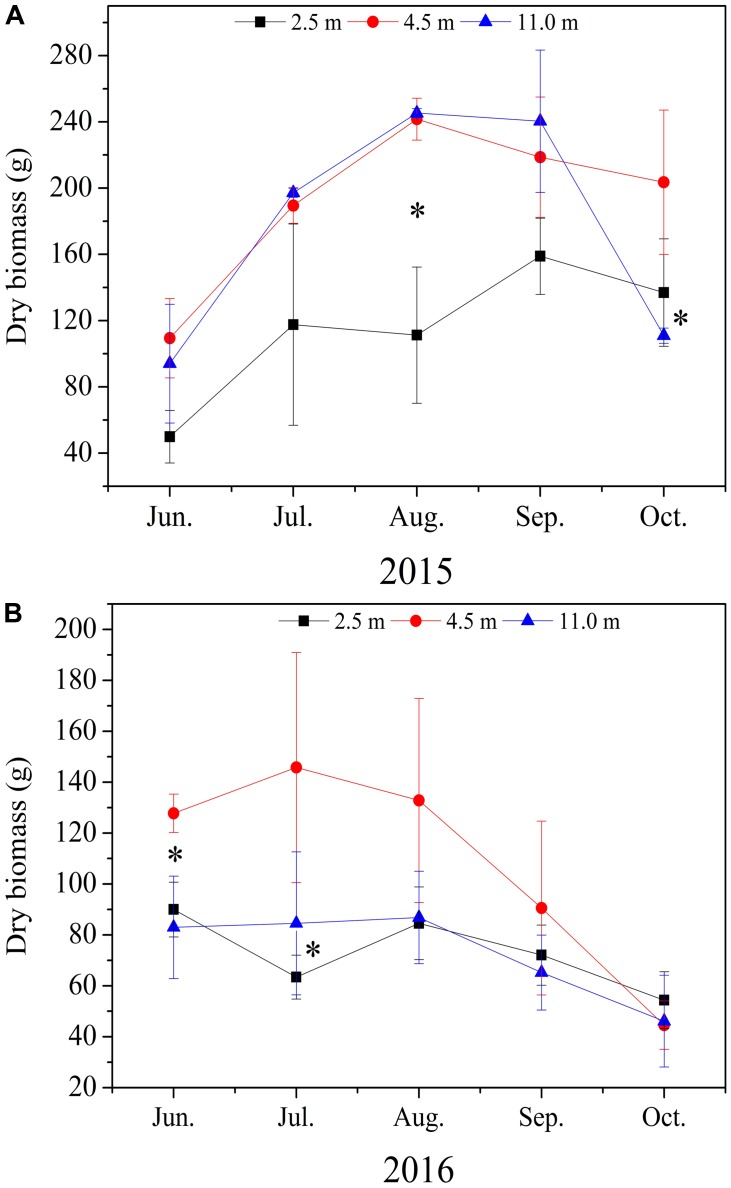
Variations in dry biomass of *Alhagi sparsifolia* at varying groundwater depths of 2.5, 4.5, and 11.0 m in 2015 **(A)** and 2016 **(B)** growing seasons. The vertical bars are standard deviations, and ^∗^ indicates significant difference at *p* < 0.05 level among the different groundwater depths.

Groundwater depth remarkable affected the dry biomass of *A. sparsifolia* (**Figure 1**). In 2015 and 2016, dry biomass of *A. sparsifolia* at 2.5 m was significantly lower than those at 4.5 and 11.0 m (**Figures 1A,B**).

### Soil C, N, and P Stoichiometric Patterns Influenced by Groundwater Depth

**FIGURE 2:**
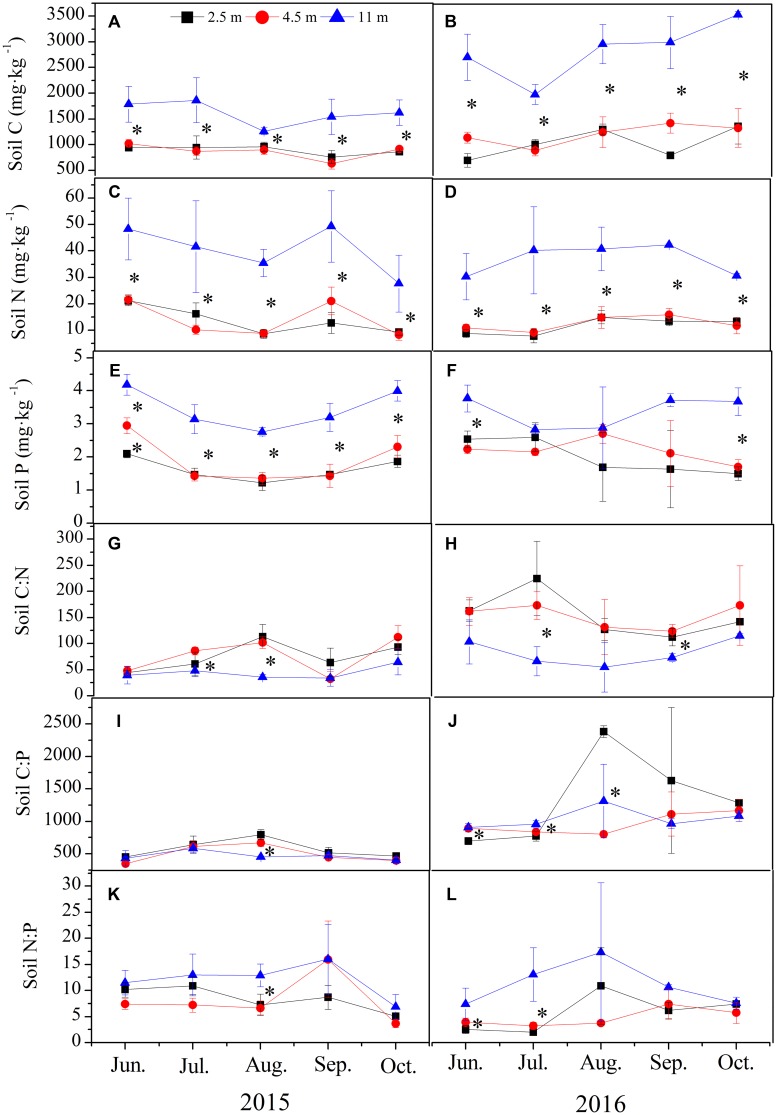
Variations in soil C, N, and P stoichiometry at varying groundwater depths of 2.5, 4.5, and 11.0 m in 2015 **(A,C,E,G,I,K)** and 2016 **(B,D,F,H,J,L)** growing seasons. The vertical bars are standard deviations, and ^∗^ indicates significant difference at *p* < 0.05 level among the different groundwater depths.

In both years, groundwater depth remarkable affected the soil C, N, and P concentrations (**Figures 2A–F**). Whereas the soil C:N, C:P, and N:P mass ratios were hardly affected by groundwater depth (**Figures 2G–L**). The soil C, N, and P concentrations at 11.0 m were significant higher than those at 2.5 and 4.5 m in both years (**Figures 2A–F**). No significant variations in these concentrations were observed between 2.5 and 4.5 m.

### Leaf C, N, and P Stoichiometric Patterns Affected by Groundwater Depth

**FIGURE 3:**
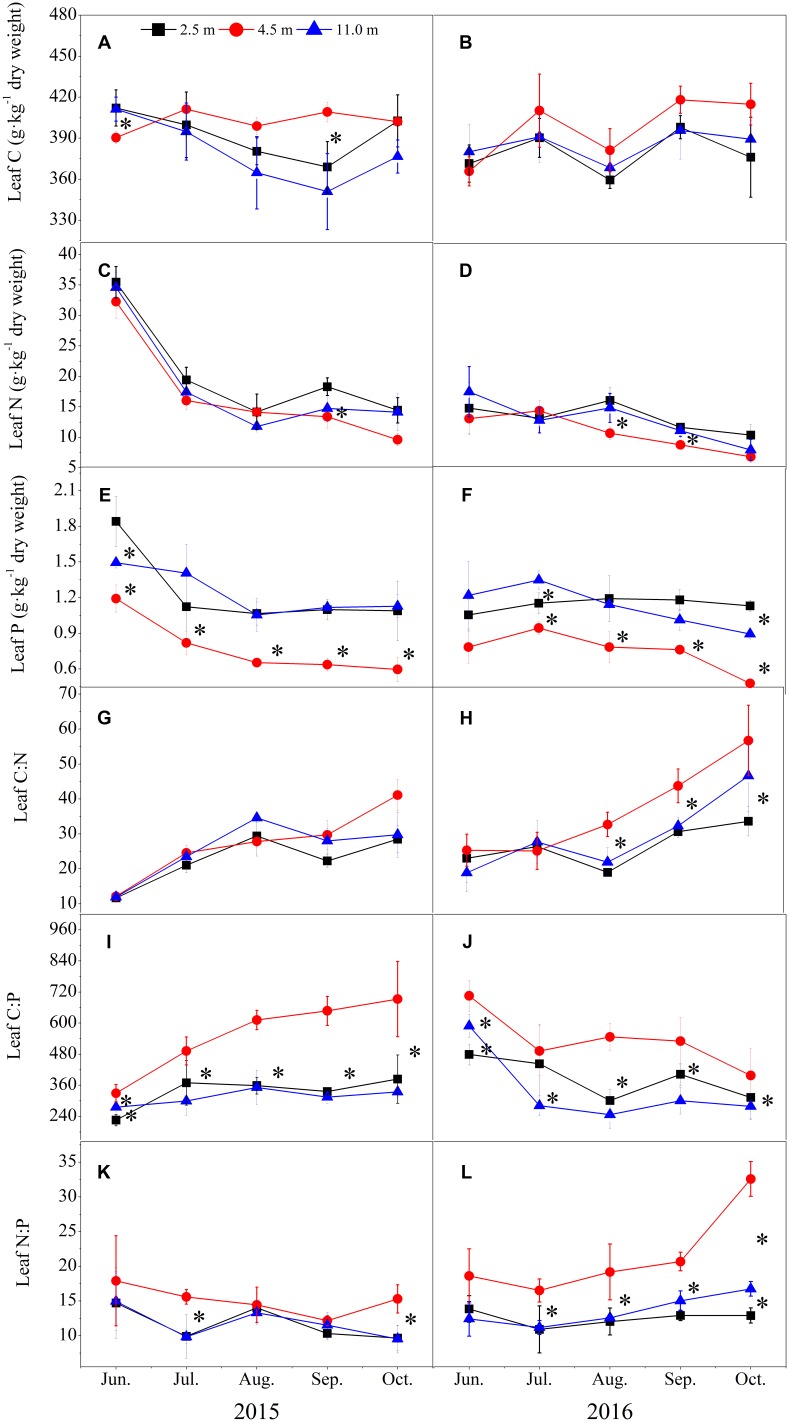
Variations in leaf C, N, and P stoichiometry of *A. sparsifolia* at varying groundwater depths of 2.5, 4.5, and 11.0 m in 2015 **(A,C,E,G,I,K)** and 2016 **(B,D,F,H,J,L)** growing seasons. The vertical bars are standard deviations, and ^∗^ indicates significant difference at *p* < 0.05 level among the different groundwater depths.

In both years, the groundwater depth significantly affected the leaf P concentration and C:P and N:P mass ratios, but it did not influence the leaf C, N concentrations and C:N mass ratio in *A. sparsifolia* (**Figure 3**). The leaf P concentration at 4.5 m was significantly lower than those at 2.5 and 11.0 m (**Figures 3E,F**). By contrast, the leaf C:P and N:P mass ratios at 4.5 m were significantly higher than those at 2.5 and 11.0 m (**Figures 3I–L**).

The leaf N and P concentrations of *A. sparsifolia* at all groundwater depths were the highest in June, but these concentrations significantly decreased from July to October (**Figures 3C–F**). The leaf C:N and C:P mass ratios increased as the growing season continued (**Figures 3G–J**). The leaf C and N:P mass ratios were relatively stable in the growing seasons in 2015 and 2016 (**Figures 3A,B,K,L**).

### Correlation of Soil C, N, and P Stoichiometry and Leaf C, N, and P Stoichiometry

Soil C concentration was positively correlated with soil N and P concentrations in both years (**Table 1**). The soil N concentration was correlated positively with soil P concentration. However, the relationship between soil and plant stoichiometric parameters was generally insignificant, but soil P concentration was positively correlated with leaf P concentration in 2015 (**Table 2**). For example, the plant C and N concentrations were not related to their respective concentrations in soils. Soil N concentration was positively correlated with leaf P concentration, and soil C:N mass ratio was negatively correlated with leaf P concentration in 2015 and 2016 (**Table 2**).

**Table 1:** Correlation of soil C, N, and P stoichiometry.

2015	Soil C	Soil N	Soil P	Soil C:N	Soil C:P	Soil N:P
Soil C	1					
Soil N	0.700^∗∗^	1				
Soil P	0.821^∗∗^	0.727^∗∗^	1			
Soil C:N	-0.261	-0.745^∗∗^	-0.425^∗∗^	1		
Soil C:P	-0.030	-0.301^∗^	-0.542^∗∗^	0.456^∗∗^	1	
Soil N:P	0.182	0.719^∗∗^	0.121	-0.780^∗∗^	-0.037	1

**2016**	**Soil C**	**Soil N**	**Soil P**	**Soil C:N**	**Soil C:P**	**Soil N:P**

Soil C	1					
Soil N	0.766^∗∗^	1				
Soil P	0.612^∗∗^	0.467^∗∗^	1			
Soil C:N	-0.454^∗∗^	-0.784^∗∗^	-0.200	1		
Soil C:P	-0.095	-0.012	-0.571^∗∗^	-0.150	1	
Soil N:P	-0.017	0.146	-0.475^∗∗^	-0.286	0.978^∗∗^	1

*^∗∗^ and ^∗^ indicate the correlation is significant at 0.01 and 0.05 level, respectively (Pearson correlation method, two-tailed).*

**Table 2:** Correlation of C, N, and P stoichiometry of *A. sparsifolia* and soil C, N, and P stoichiometry.

2015	Soil C	Soil N	Soil P	Soil C:N	Soil C:P	Soil N:P
C	-0.151	-0.288	-0.146	0.125	-0.009	-0.244
N	0.170	0.267	0.307^∗^	-0.385^∗∗^	-0.240	0.103
P	0.393^∗∗^	0.413^∗∗^	0.426^∗∗^	-0.412^∗∗^	-0.194	0.157
C:N	-0.101	-0.229	-0.151	0.405^∗∗^	0.073	-0.181
C:P	-0.427^∗∗^	-0.429^∗∗^	-0.400^∗∗^	0.359^∗^	0.072	-0.177
N:P	-0.230	-0.097	-0.075	-0.126	-0.109	0.001

**2016**	**Soil C**	**Soil N**	**Soil P**	**Soil C:N**	**Soil C:P**	**Soil N:P**

C	-0.103	-0.086	0.029	-0.067	-0.178	-0.174
N	-0.042	0.074	0.108	0.002	-0.075	-0.066
P	0.144	0.372^∗^	0.141	-0.316^∗^	-0.041	0.027
C:N	0.076	-0.071	-0.058	0.006	0.018	0.003
C:P	-0.140	-0.277	-0.157	0.251	-0.015	-0.065
N:P	-0.192	-0.272	-0.067	0.318^∗^	-0.047	-0.100

*^∗∗^ and ^∗^ indicate the correlation is significant at 0.01 and 0.05 level, respectively (Pearson correlation method, two-tailed).*

## Discussion

### Biomass and Soil Available Nutrients Were Affected by Groundwater Depth

In our study, the biomass of *A. sparsifolia* increased at 4.5 m compared with that at 2.5 m, whereas this parameter decreased at 11.0 m (**Figures 1A,B**). This result is in agreement with the findings of Peng et al. (2014), who reported that the biomass of *A. sparsifolia* in an artificial simulation groundwater depth increases from 0.4 to 1.2 m and decrease at 1.8 and 2.2 m. These results are also consistent with the observation that groundwater is the major source of nutrients and water for *A. sparsifolia* (Arndt et al., 2004; Zeng et al., 2006), thereby confirming that the change in groundwater depths can remarkable affect the growth of *A. sparsifolia.* Therefore, if the groundwater resource of Cele oasis continuously declines, then the growth of *A. sparsifolia* may be severely inhibited.

In this study, the soil C, N, and P concentrations at 11.0 m were significantly higher than those at 2.5 and 4.5 m in 2015 and 2016 (**Figures 2A–F**). This result is consistent with previous findings, which revealed an increase in soil N concentration with declining groundwater depth in dune slack soils (Rhymes et al., 2016). An increase in soil N concentration at deep groundwater depth is likely caused by a reduction in soil moisture content, which can inhibit denitrification rates, leading to increased N retention and eutrophication effect (Rhymes et al., 2016). Similarly, we believed that dry soil with declining groundwater depth affects the movement and cycle of soil C and P, resulting in increased retention and eutrophication effect in soil C and P. Hence, soil C and P concentrations were higher at 11.0 m than at 2.5 and 4.5 m.

### Leaf P, But Not C and N Concentrations Were Affected by Groundwater Depth

The leaf P concentration and the C:P and N:P mass ratios in *A. sparsifolia* were significantly affected by groundwater depths in both years (**Figure 3**). This results is similar to previous findings, indicating that the leaf P concentrations and C:P and N:P mass ratios of the submersed macrophytes are affected by increasing groundwater depths (Li W. et al., 2015). The effect of groundwater depth on leaf P concentration is likely attributed to soil and groundwater that serve as the only sources of P absorbed by *A. sparsifolia* in the current study; thus this phenomenon can be significantly affected by external environments (Hedin, 2004; Rong et al., 2015). Our results are similar to the observation of Zhang et al. (2018), who demonstrated that soil P concentration significantly affects the growth of *A. sparsifolia* in deserts. By contrast, our previous studies revealed that high ${\text{NO}}_{3}^{\text{–}}$ and ${\text{PO}}_{3}^{2\text{–}}$ concentrations in the xylem sap of *A. sparsifolia* indicate sufficient nutrient supply through groundwater (Arndt et al., 2004; Zeng et al., 2006). However, in the present study, the soil P concentration also showed significant influenced the P uptake of *A. sparsifolia* in 2015. Therefore, the soil and groundwater depth acted as sources of P absorbed by *A. sparsifolia*. The leaf P concentration at 4.5 m was obviously depressed, corresponding with a lower soil N concentration. This result is consistent with that of Wang et al. (2015), who found that shortage in N availability hinders P uptake from soils into plants.

Leaf C concentration in *A. sparsifolia* was relatively stable at 400 g kg^-1^ regardless of the groundwater depth. Our results are consistent with previous findings, which demonstrated that the C concentration of *T. chinensis*, a desert plant, is also stable in response to environmental stresses (Rong et al., 2015). Stable C in plant tissues is due to C accumulation determined by C fixation through photosynthesis (Wang et al., 2015). Hence, for leaf C concentration, photosynthesis is more important than soil nutrient concentration. Similar to C concentration, the N concentration of *A. sparsifolia* was not affected by groundwater depth. N seemed not being a limiting growth factor for *A. sparsifolia* to adapt to declining groundwater depth in a desert habitat because *A. sparsifolia* is a leguminous plant that can fix atmospheric N_2_. This result is consistent with the previous findings, which indicated N_2_ fixation from air contributes more than 80% of the total leaf N for almost half of *A. sparsifolia* in the Taklamakan Desert (Arndt et al., 2004).

In this study, the C:P and N:P mass ratios were affected by groundwater depth because of the changes in P concentration in *A. sparsifolia*. C:N and C:P mass ratios reflect the ability of plants to assimilate C when they simultaneously absorb N and P (Koerselman and Meuleman, 1996; Aerts and Chapin, 1999; Güsewell, 2004; He et al., 2008; Rong et al., 2015). Here, the leaf C:P mass ratio increased at 4.5 m and then decreased at 11.0 m, suggesting that the significant reduction of groundwater level may inhibit the ability of plants to absorb P. Previous studies suggested that the leaf N:P ratio is a vital indicator of the limiting nutrient elements of plants and widely used in a series of ecological stoichiometric studies (Koerselman and Meuleman, 1996; Tessier and Raynal, 2003; Güsewell, 2004; Wu et al., 2012). For example, Güsewell (2004) considered that a N:P mass ratio lower than 10 indicates N limitation for terrestrial plants, while a N:P mass ratio greater than 20 corresponds to P limitation. Zhang et al. (2004) suggested that a N:P mass ratio lower than 21 represents N limitation, while a mass N:P ratio greater than 23 shows P limitation. However, in wetland ecosystems, plant growth is limited by P when N:P > 16 and restricted by N when N:P < 14. Plant growth is also limited by restricted by N and P when 14 < N:P < 16 (Koerselman and Meuleman, 1996; Wu et al., 2012). In the present study, the leaf N:P mass ratio of *A. sparsifolia* at 2.5, 4.5, and 11.0 m ranged between 13.20 and 19.34, between 16.53 and 27.15, and between 11.34 and 23.14 in 2015, respectively, and between 9.64 and 14.70, between 12.15, and 17.90, and between 9.51 and 14.96 in 2016, respectively. These results suggested neither N nor P was a limiting factor of *A. sparsifolia* growth at different groundwater depths. This result was consistent a previous finding, which revealed that *A. sparsifolia* is a leguminous plant that can fix atmospheric N_2_ from the atmosphere; atmospheric N_2_ fixation contributes more than 80% of the total leaf N for almost half of *A. sparsifolia* in the Taklamakan Desert (Arndt et al., 2004). Therefore, *A. sparsifolia* was not limited by N in its habitat.

The leaf N:P mass ratio of *A. sparsifolia* at 4.5 m was significantly higher than those at 2.5 and 11.0 m (**Figures 3I–L**). The leaf P concentration at 4.5 m was significantly lower than those at 2.5 and 11.0 m (**Figures 3E,F**). These results suggested that the growth of *A. sparsifolia* at 4.5 m was affected by P concentration likely because P concentration in *A. sparsifolia* at 4.5 m might be diluted by increased plant biomass (**Figure 1**). This assumption is supported by the fact that soil P concentration at 4.5 m was similar to that at 2.5 m. Moreover, this result is consistent with a previous finding, which revealed that soil P concentration significantly affects the growth of *A. sparsifolia* in deserts (Zhang et al., 2018).

### Relationship Between Soil Nutrient and Plant C, N, and P Stoichiometry

Although previous studies found that significantly positive correlations exist between soil nutritional status and plant nutrient concentration, these studies did not conclude a solid relationship between soil nutrient concentration and plant C, N, and P stoichiometry (Wang et al., 2015; Zeng et al., 2017). In our study, a positive relationship was observed between soil N and P concentrations of *A. sparsifolia* (**Table 2**), suggesting that the increased level of soil N may have increased the uptake of N in plants; as a result, *A. sparsifolia* absorbs more P because of elemental homeostasis in plants (Elser et al., 2000; Cross et al., 2005; Rong et al., 2015; Zhan et al., 2017). Therefore, in our study, soil N was a key factor that affected the C, N, and P stoichiometry of *A. sparsifolia* under different groundwater depths because soil N concentration was also significantly correlated with soil P concentration and C:P mass ratio (**Table 1**). Furthermore, this result was supported by previous findings, indicating that soil P concentration is positively associated with soil N (Cleveland and Liptzin, 2007; Hume et al., 2016), and shortage of N availability in soil affects the P uptake of plants (Wang et al., 2015). Therefore, groundwater depths significantly influenced soil nutrients, especially soil N, resulting in variations in the leaf P concentration, C:P mass ratio of *A. sparsifolia* because of the interactive relationships of soil C, N, and P. In contrast to previous studies, our study demonstrated that the source of nutrients absorbed by *A. sparsifolia* was not only groundwater but also soil.

### Adaptive Strategies: Defensive or Competitive?

Previous studies showed that competitive and defensive life strategies are determined by the changes in C:N and C:P mass ratios (Rong et al., 2015). A higher plant growth rate is mainly accompanied by low C:N and C:P mass ratios. These two parameters are also effective in reflecting plant growth status and health condition; for example, a rapid growth rate is strongly correlated with decreased C:P mass ratio (Elser et al., 2000; Sterner and Elser, 2002). Rong et al. (2015) reported that high C:N and C:P mass ratios in the middle stage of *T. chinensis* growth indicate that a defensive strategy of plants grown in coastal wetlands because of high temperatures, intensive soil evaporation, and plant transpiration. In our study, the leaf C:N and C:P mass ratios of *A. sparsifolia* at 4.5 m were significantly higher than those at 2.5 and 11.0 m (**Figures 3G,J**), suggesting that *A. sparsifolia* at 4.5 m probably employed a defensive strategy. This result is also consistent with the finding that the largest biomass (**Figure 1**) and lowest leaf P concentration of *A. sparsifolia* were observed at 4.5 m among the various groundwater depths (**Figures 3E,F**). *A. sparsifolia* at 2.5 and 11.0 m groundwater depths probably undertook a competitive strategy because of the lower leaf C:N and C:P ratios (**Figures 3G,J**), lower biomass of *A. sparsifolia* (**Figure 1**), and higher leaf P concentration (**Figures 3E,F**) than those at 4.5 m.

Further specific studies are needed to investigate the efficiency of the resorption and utilization of C, N, and P at the declining groundwater depth. This study helps facilitate the ecological conservation and environmental protection of the desert-oasis ecotone of the southern rim of the Taklamakan Desert in China.

## Conclusion

Groundwater depth significantly influenced the biomass, soil C, N, and P concentrations, and leaf P concentration of *A. sparsifolia*. *A. sparsifolia* had larger biomass and lower leaf P concentration at 4.5 m than at 2.5 and 11.0 m groundwater depths. The soil C, N, and P concentrations at 4.5 m were significantly lower than those at 11.0 m. The soil N concentration was significantly and positively correlated with leaf P concentration at all of the groundwater depths. The leaf P concentration of *A. sparsifolia* was lower at 4.5 m than at 2.5 and 11.0 m likely because of a biomass dilution effect. By contrast, the leaf C and N concentrations were generally unaffected by groundwater depth, confirming that C and N accumulations in *A. sparsifolia* were predominantly determined by C fixation through photosynthesis and biological atmospheric N_2_ fixation, respectively. As a result, the C:N and C:P mass ratios of *A. sparsifolia* at 4.5 m were higher than those at the groundwater depths, implying a defensive life history strategy. Conversely, *A. sparsifolia* likely adopted a competitive strategy at 2.5 and 11.0 m as indicated by the lower C:N, and C:P mass ratios that those at 4.5 m. Our results could facilitate the understanding of the C, N, and P stoichiometry of desert phreatophytes and their responses to the declining groundwater depth in the desert-oasis ecotone of northwest China. This study could further enrich the existing stoichiometric studies.

## Author Contributions

BZ and FZ: conception and design, analysis and interpretation and critical revision of the article, and overall responsibility; XG and MS: revise manuscript; LL, YL, GL, and CH: data analysis; DG: data collection.

## Conflict of Interest Statement

The authors declare that the research was conducted in the absence of any commercial or financial relationships that could be construed as a potential conflict of interest.
